# Dataset for evaluating fitness index using Adaptive Neuro-Fuzzy Inference System

**DOI:** 10.1016/j.dib.2018.07.049

**Published:** 2018-08-04

**Authors:** Chaitanya Kulkarni, Soham Kulkarni, Jayakrishna Kandasamy

**Affiliations:** School of Mechanical Engineering, VIT University, Vellore, India

## Abstract

With the current global downturn, the organizations need to develop new strategies and innovative approaches to ensure that every aspect of sustainability is achieved. For this purpose, the organizations need an indicator that measures the fitness if an organization. The purpose of this project is to analyze the ‘Fitness’ of an organization using the dataset related to leanness, agility and sustainability in ANFIS (Adaptive Neuro-Fuzzy Inference System) in order to determine whether the company is fit enough to sustain in global markets or not. The project does so by integrating both neural networks and fuzzy logic principles with lean, agility and sustainability principles. FIT manufacturing is the integration of Lean, Agile and sustainability manufacturing in one system as a whole which would help in attaining maximum output and sustain effectively in global markets. FIT Manufacturing adopts an integrated approach towards the use of Lean, Agility and Sustainability to achieve a level of fitness that is unique to each company. The database in the paper contains lean, agile and sustainable indices reviewed by experts. FIT does not prescribe that every aspect of Lean, Agile and Sustainability methodologies must be applied to every company, but a selective mix of components will provide the optimum conditions for a company to prosper.

**Specifications Table**

**Table:** 

Subject area	Industrial Engineering
More specific subject area	Fit manufacturing
Type of data	Table, image, graph, figure
How data was acquired	Industrial survey was conducted
Data format	analyzed
Experimental factors	Lean, Agile and Sustainable manufacturing criteria were established and collected data was compiled in form of a matrix.
Experimental features	The fuzzy logic designer of MATLAB was used for calculation of Agility Index, Lean Index and Sustainability index. Based on these values, FITNESS index was determined.
Data source location	Automobile component manufacturing company located in Tamil Nadu, India
Data accessibility	All the data is available at https://data.mendeley.com/datasets/2ytrmrjztt/1

**Value of the data**

• Markets all over the globe are getting more and more competitive through the changes driven by emerging technologies which creates the need for an organization to be adaptive and compatible in order to dominate the market.
• FIT manufacturing is a perfect amalgamation of core principles of lean, agile and sustainable manufacturing.
• The data in this article helps to determine lean, agile and sustainability indices which provides an insight as to how to fit the organization is by providing a framework for the calculation of fitness index.
• Using this data, the efficiency and responsiveness of an organization towards the constantly changing markets can be analyzed.

## 1 Data

This dataset consists of 5 fitness enablers, 69 fitness criteria and 205 fitness attributes. The data consists of all major dimensions or enablers of fitness such as management responsibility, manufacturing management, manufacturing technology, workforce and manufacturing strategy. The data on agile, lean and sustainability criteria, was collected from automobile components manufacturing company located in Tamil Nadu, India arranged in a matrix form (Table 1, Table 2, Table 3, Table 4, Table 5).

**Table 1:** Agility criteria matrix.

**Table 2:** Lean criteria matrix.

**Table 3:** Social Sustainability criteria matrix.

**Table 4:** Economical Sustainability criteria matrix.

**Table 5:** Environmental Sustainability criteria matrix.

## 2 Experimental design, materials and methods

The methodologies that allowed the data presented are described in [1], [2] and in cited references.

### 2.1 Data collection

The data presented in the paper was collected from automobile components manufacturing company located in Tamil Nadu. India. The attributes pertaining to leanness, agility and sustainability criteria were obtained and rated on a scale from 1 to 10 by industry experts.

### 2.2 Rules for criterions

I. If (agility is not_agile) and (leanness is not_lean) and (sustainability is not_sustainable) then (fitness is not_fit) (1)
II. If (agility is average) and (leanness is average) and (sustainability is average) then (fitness is avergge) (1)
III. If (agility is agile) and (leanness is lean) and (sustainability is sustainable) then (fitness is fit) (1)
IV. If (agility is agile) and (leanness is average) and (sustainability is sustainable) then (fitness is fit) (1)
V. If (agility is agile) and (leanness is average) and (sustainability is average) then (fitness is avergge) (1)
VI. If (agility is average) and (leanness is lean) and (sustainability is average) then (fitness is avergge) (1)
VII. If (agility is average) and (leanness is lean) and (sustainability is sustainable) then (fitness is fit) (1)
VIII. If (agility is average) and (leanness is average) and (sustainability is sustainable) then (fitness is fit) (1)
IX. If (agility is average) and (leanness is average) and (sustainability is not_sustainable) then (fitness is avergge) (1)
X. If (agility is average) and (leanness is not_lean) and (sustainability is not_sustainable) then (fitness is not_fit) (1)
XI. If (agility is not_agile) and (leanness is average) and (sustainability is not_sustainable) then (fitness is not_fit) (1)
XII. If (agility is not_agile) and (leanness is average) and (sustainability is average) then (fitness is avergge) (1)
XIII. If (agility is average) and (leanness is average) and (sustainability is not_sustainable) then (fitness is avergge) (1)
XIV. If (agility is not_agile) and (leanness is average) and (sustainability is sustainable) then (fitness is avergge) (1)
XV. If (agility is agile) and (leanness is average) and (sustainability is not_sustainable) then (fitness is avergge) (1)
XVI. If (agility is agile) and (leanness is not_lean) and (sustainability is sustainable) then (fitness is avergge) (1)
XVII. If (agility is agile) and (leanness is not_lean) and (sustainability is not_sustainable) then (fitness is not_fit) (1)
XVIII. If (agility is not_agile) and (leanness is not_lean) and (sustainability is sustainable) then (fitness is not_fit) (1)
XIX. If (agility is not_agile) and (leanness is lean) and (sustainability is not_sustainable) then (fitness is not_fit) (1)
XX. If (agility is not_agile) and (leanness is lean) and (sustainability is not_sustainable) then (fitness is not_fit) (1)

### 2.3 Matrices Index Evaluation by means of Adaptive Neuro-Fuzzy Inference System

**Image 1:**
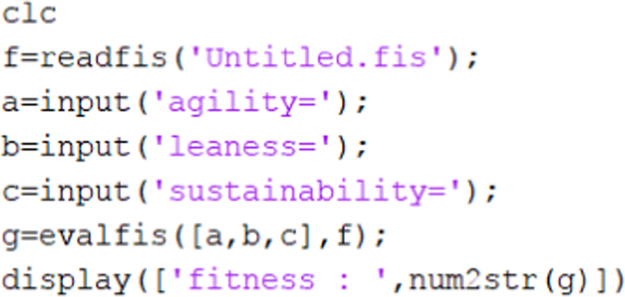
MATLAB code used for data evaluation.

**Fig. 1:**
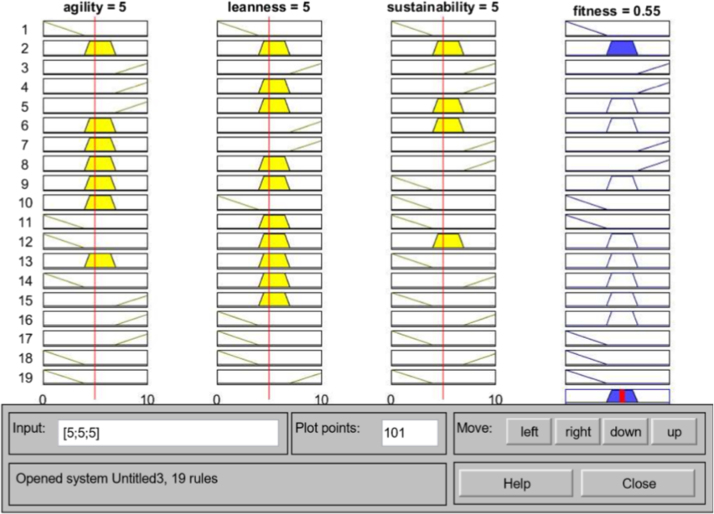
Rule viewer interface for leanness, agility and sustainability criteria.

**Fig. 2:**
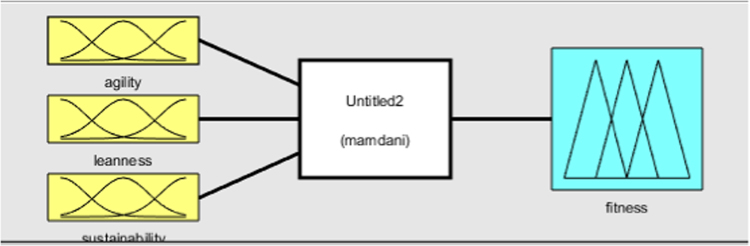
Fuzzy logic designer interface and Membership function input.

**Fig. 3:**
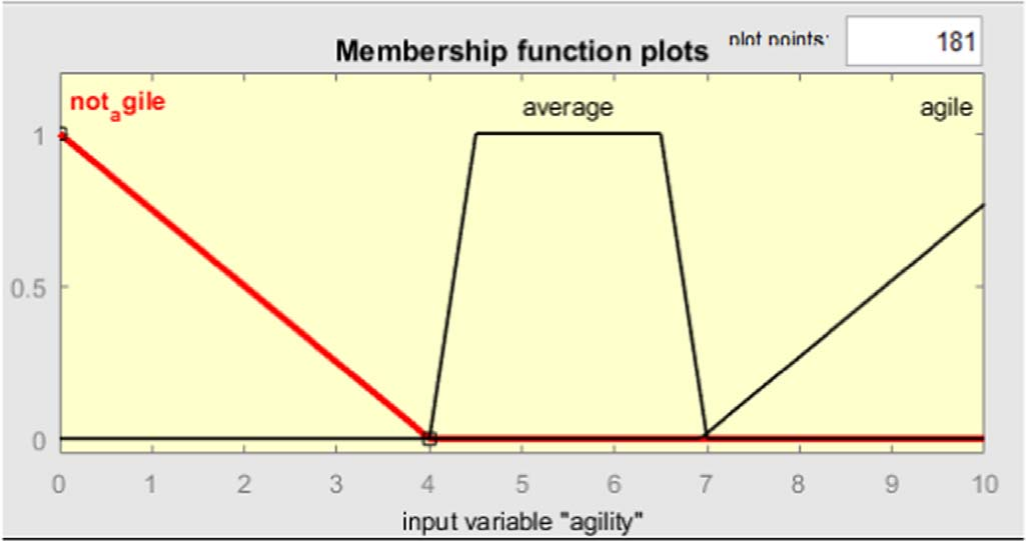
Membership function of agility criterion.

**Fig. 4:**
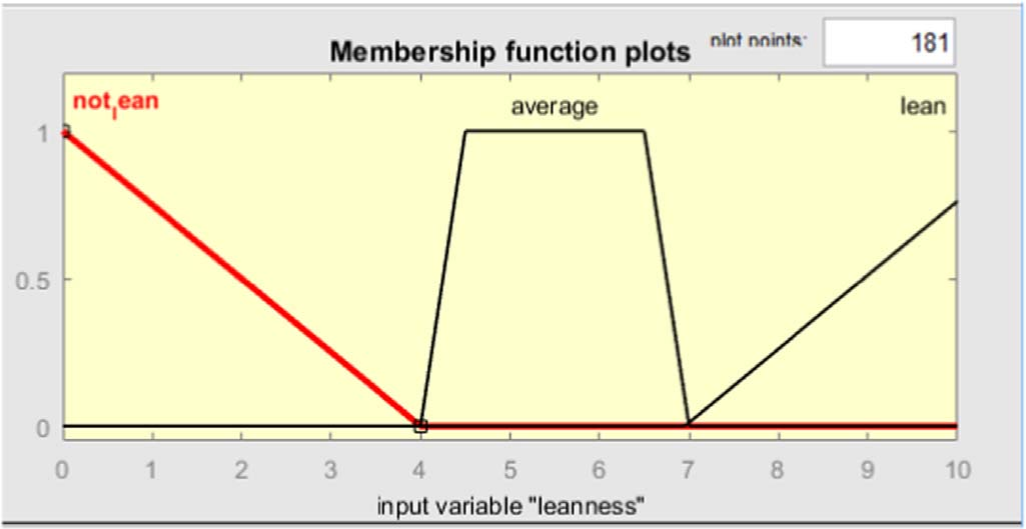
Membership function of leanness criterion.

**Fig. 5:**
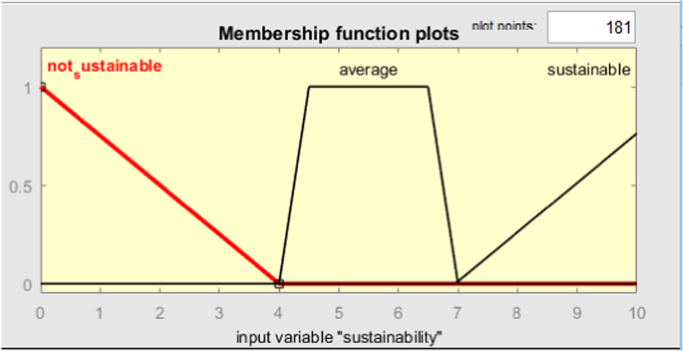
Membership function of sustainability criterion.

MATLAB 2016 was used for creation of Adaptive Neuro-Fuzzy Inference System. The ANFIS model was created using fuzzy logic designer with the corresponding matrices as input (Image 1 and Fig. 1, Fig. 2, Fig. 3, Fig. 4, Fig. 5).

### 2.4 3d surfaces from fuzzy logic output

**Fig. 6:**
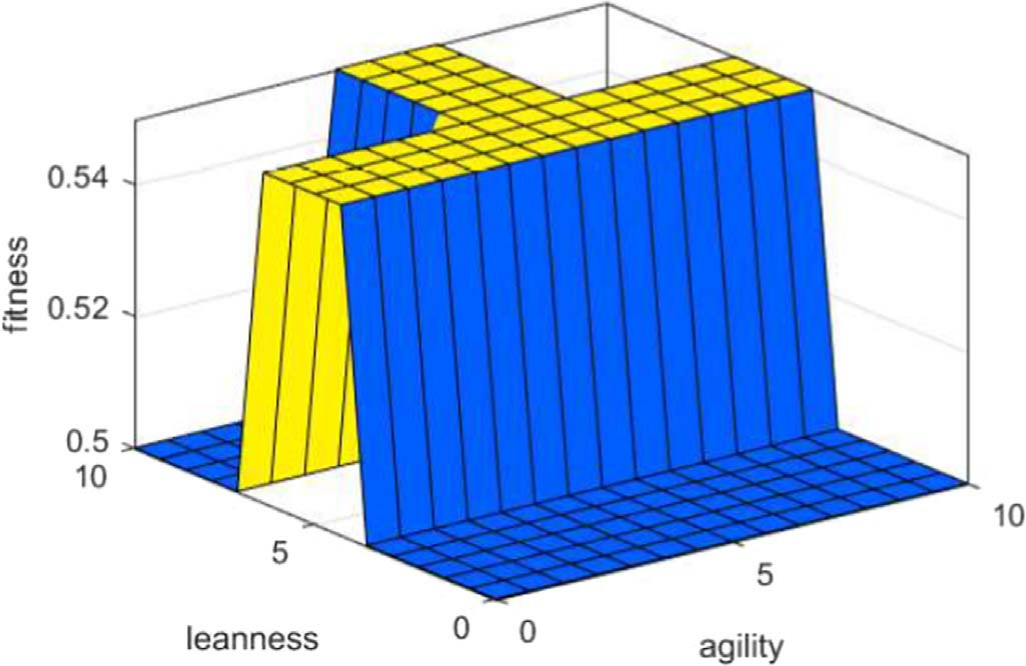
X(input): agility Y (input): leanness Z (output): fitness.

**Fig. 7:**
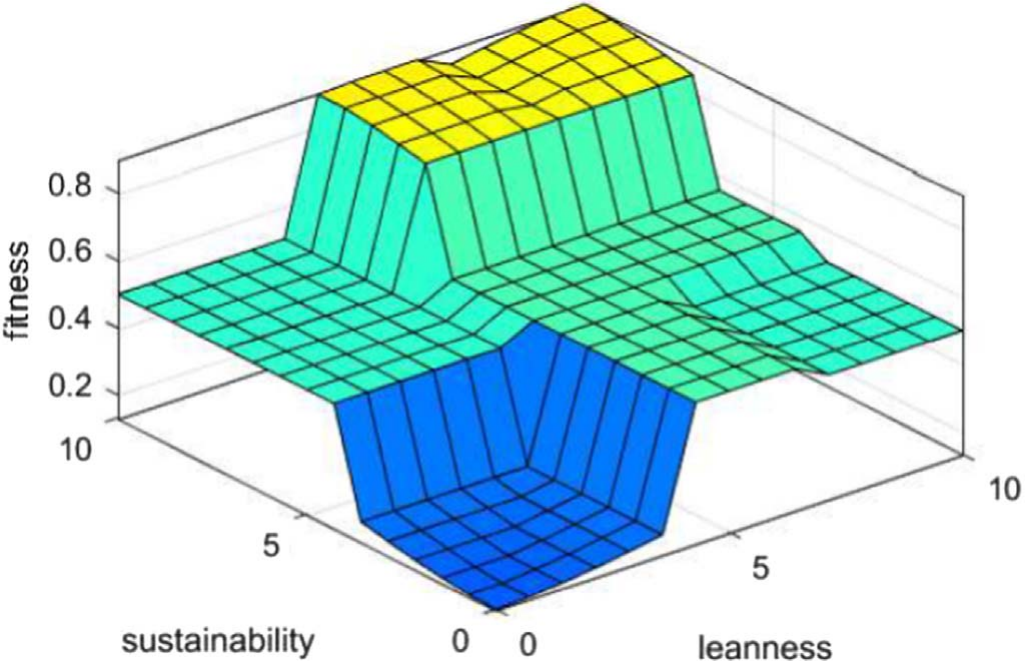
X(input): leanness Y (input): sustainability Z (output): fitness.

**Fig. 8:**
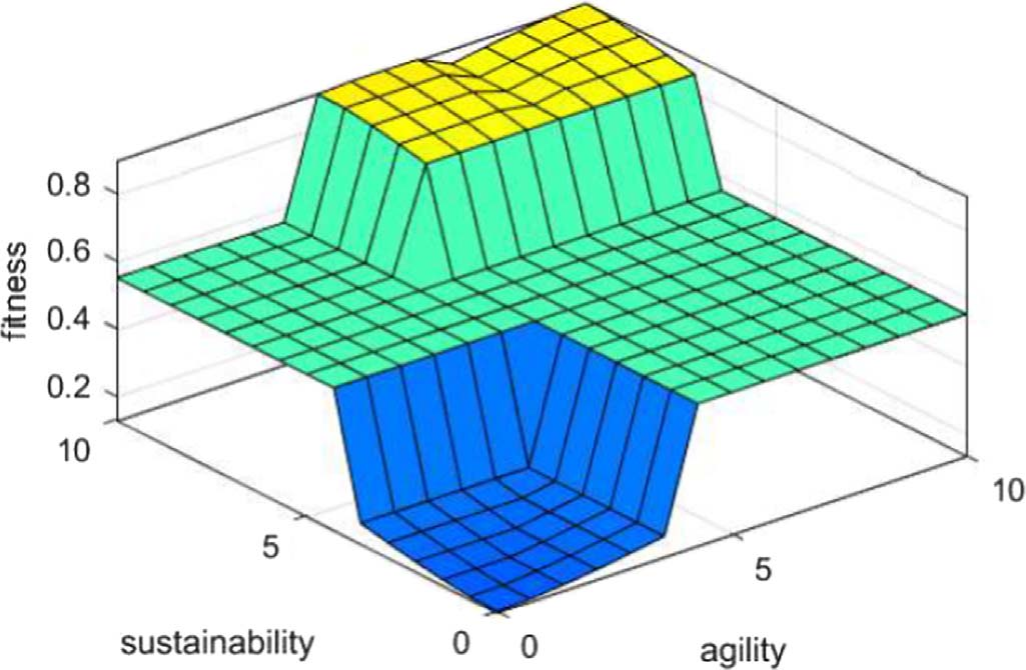
X(input): agility Y (input): sustainability Z (output): fitness.

See Fig. 6, Fig. 7, Fig. 8.

## 3 Scope

FIT manufacturing is the integration of Lean, Agile and sustainability manufacturing in one system which would help in attaining maximum output and prevent the damage caused to the environment. This paper deals with analyzing the ‘fitness’ of a company using ANFIS. The integration of the Fit principles can be employed to reduce the unnecessary cost and increase efficiency of production with less wastage in a market having mercurial demand patterns. The fitness index can be used as a benchmark to evaluate overall efficiency of an organisation.
